# Coupling Artificial Intelligence with Proper Mathematical Algorithms to Gain Deeper Insights into the Biology of Birds’ Eggs

**DOI:** 10.3390/ani15030292

**Published:** 2025-01-21

**Authors:** Valeriy G. Narushin, Natalia A. Volkova, Alan Yu. Dzhagaev, Darren K. Griffin, Michael N. Romanov, Natalia A. Zinovieva

**Affiliations:** 1 Research Institute for Environment Treatment, 69032 Zaporizhya, Ukraine; val@vitamarket.com.ua; 2 Vita-Market Ltd., 69032 Zaporizhya, Ukraine; 3 L. K. Ernst Federal Research Centre for Animal Husbandry, Dubrovitsy, Podolsk 142132, Moscow Oblast, Russia; natavolkova@inbox.ru (N.A.V.); alan_dz@inbox.ru (A.Y.D.); 4 School of Natural Sciences, University of Kent, Canterbury CT2 7NJ, Kent, UK; d.k.griffin@kent.ac.uk; 5 Animal Genomics and Bioresource Research Unit (AGB Research Unit), Faculty of Science, Kasetsart University, Chatuchak, Bangkok 10900, Thailand

**Keywords:** deep learning (DL), artificial intelligence (AI), machine learning (ML), avian egg studies, egg shape models, egg mathematical indices, egg volume, egg surface area, prediction of egg inner parameters, non-destructive testing

## Abstract

Avian eggs are products of consumer demand, with modern methodologies for their morphometric analysis used for improving quality, productivity and marketability. Such studies open up numerous prospects for the introduction of artificial intelligence (AI) and deep learning (DL). We first consider the state of the art of DL in the poultry industry, e.g., image recognition and applications for the detection of egg cracks, egg content and freshness. We comment on how algorithms need to be properly trained and ask what information can be gleaned from egg shape. Considering the geometry of egg profiles, we revisit the Preston–Biggins egg model, the Hügelschäffer’s model, universal egg models, principles of egg universalism and “The Main Axiom”, proposing a series of postulates to evaluate the legitimacy and practical application of various mathematical models. We stress that different models have pros and cons, and using them in combination may yield more useful results than individual use. We consider the classic egg shape index alongside other alternatives, drawing conclusions about the importance of indices in the context of applying DL going forward. Examining egg weight, volume, surface area and air cell calculations, we consider how DL might be applied, e.g., for egg storage. The value of DL in egg studies is in pre-incubation egg sorting, the optimization of storage periods and incubation regimes, and the index representation of dimensional characteristics. Each index can thus be combined to provide a synergy that is on the threshold of many scientific discoveries, technological achievements and industrial successes facilitated through AI and DL.

## Simple Summary

Most birds’ eggs are products of consumer demand (for direct consumption or used to produce edible birds, mostly chickens). Many scientists are thus engaged in analyzing their shape to help improve quality, productivity and marketability, and these studies open up prospects for the use of artificial intelligence (AI). Deep learning (DL) is a form of AI concerned with computers taking data and extrapolating them to new ideas without direct human guidance. DL is based on the workings of animal brains, and specifically performs classification, analysis and further scholarly tasks by learning from existing datasets. We first consider the “state of the art” of DL in the poultry industry, including image recognition and applications for detecting cracks, egg content and freshness. We comment on how AI algorithms need to be properly trained and consider egg profile geometry. We revisit previous publications’ egg shape mathematics, commenting on the pros/cons of each. Examining weight, volume, surface area and air cell calculations, we consider how DL might be applied to these. The future value of DL is in egg sorting before incubation, storage/incubation and dimension calculation. Combining mathematical models with AI/DL means that we are on the threshold of many scientific discoveries, technological achievements and industrial successes.

## 1. Introduction

Most commercial avian eggs are products of increasing consumer demand and consumption [1,2], with recent significant production rises associated with the expanding profitability of the table egg and poultry meat industries [3,4,5]. Modern methodologies for the morphometric analysis of bird eggs, their non-invasive assessment and the development of mathematical indices and methods for predicting egg quality (e.g., [6]) continue to contribute to an increase in egg yield and productivity. This is particularly true when they are combined with highly marketable nutritional and incubation characteristics [7,8,9,10,11,12].

The development and application of non-destructive technologies for assessing the quality of bird eggs [13,14] opens up numerous prospects for the introduction of new approaches based on artificial intelligence (AI) and deep learning (DL) [15,16,17]. In this context, the tasks of developing new mathematical models for the non-invasive evaluation of egg parameters and mathematical formulae for calculating these parameters [18] are growing in importance.

This review focuses on promising research directions in the development of mathematical methods for studying and predicting various aspects of egg quality based on a full range of external and internal egg parameter data. It highlights those that have already begun to find applications in combination with the latest technological advances in DL and AI.

## 2. Deep Learning in Eggs: The Current State of the Art

### 2.1. Egg Image Recognition by AI and DL–Initial Studies

Deep learning (DL) entered public consciousness with the development of artificial intelligence (AI), neural networks (NNs) and machine learning (ML) algorithms [19,20,21] (for more detailed term definitions, see Table 1). The main advantage of this technique is that NNs, which are the basis of DL, independently identify patterns and adapt to training data [22,23]. This allows them to process new datasets effectively, even if they differ from those on which training took place (e.g., [24]). Despite the fairly extensive list of possible applications of DL, NNs are the most widely applied variants of AI in image and pattern recognition (e.g., [25]).

To date, there has been a rapidly growing number of examples of effective AI and DL applications in the poultry industry [34,35,36]. For instance, Febriani et al. [37] developed the so-called Smart Egg Incubator, in which a primitive image recognition system, after the DL process, distinguished hatched chicks from eggs still incubating and demonstrated 93.7% accuracy. While distinguishing a moving animal from a static egg may not seem overly impressive, particularly at less than 94% accuracy, it is nonetheless one of the only times in which DL was used in the poultry industry, and many more followed.

A second early example of the application of DL in the poultry industry is of an egg picking robot trained to identify eggs in a complex environment using a corresponding DL algorithm [38]. Again, as a very early innovation, the effort did not achieve a particularly high accuracy for egg-picking, but stands nonetheless as a groundbreaking early study from which many more followed and at least significantly increased the speed of egg identification.

From these early steps, it was clear that the use of DL was going to grow in this aspect of poultry farming and egg-related studies in general [39,40,41,42].

### 2.2. Egg Cracks and Deeper Learning

A number of studies focused on teaching AI to visualize visible damage on the shell, including breakage, cracking or holes [43]. This was attempted, for instance, by Huang et al. [44] and Sánchez et al. [45], who achieved fairly high accuracy in identifying damaged eggs, to the level of 96.4% and 97.33%, respectively. In another similar study, Nasiri et al. [46], in addition to damage, also identified dirt on the shell in their DL program. As a result, the average (across all determined parameters) accuracy of identifying such eggs was 94.84%. Further learning systems have been implemented to identify foreign objects in combination with measuring certain parameters associated with, for example, the geometric dimensions of the egg. For example, Gayathri et al. [47] developed a deep convolutional NN system to determine the size of chicken eggs automatically. For the DL procedure, the authors used a comprehensive dataset of egg images, meticulously annotated with size labels. As a result, they achieved what might not be perceived as a particularly impressive accuracy rate over 85% on the test dataset. Like the smart incubator and the egg picker, these were the very early days of DL for the poultry industry and stand as the seminal studies upon which more accurate, later ones were based.

### 2.3. The Contents of Hatching Eggs and the Determination of Freshness

In addition to external shell damage, DL algorithms have also been used to analyze the contents of hatching eggs. For such training, the authors Geng et al. [48] used images that were acquired by an ordinary CCD camera and a general point light source. The AI was trained to tell the difference between dead/infertile eggs and fertile eggs on the fifth day of incubation. The resulting model demonstrated an identification accuracy of 99.5% and marked the progression of DL as a highly accurate means of sorting eggs to the level that would be required by the industry. A similar approach was used by Çevik et al. [49], capturing images of fertilized and unfertilized eggs during incubation and performing the DL process based on the multiple images obtained.

Nakaguchi and Ahamed [14] collected air cell images of quail eggs during storage, which they used to produce models of DL algorithms. As a result, DL object detection algorithms were able to identify non-fresh quail eggs with high accuracy. To capture air cell images, the authors used a thermal microcamera.

Şahin and Önder [50] used DL methods on photographs of eggs, measuring air cell size over 29 days of storage to determine their freshness. The egg was illuminated with a conventional light source and the photographs were taken with a digital camera. After multiple training iterations, the authors achieved an accuracy of 91.78% in determining the freshness of the egg, thereby adding to the list of applications of DL. Finally, a study by Vargas et al. [51] reported that AI can be trained to recognize the internal structures of eggs of different sizes (yolk, albumen, air cell) using tomographic images.

### 2.4. Improvements in DL Algorithms in Egg Research That Are Needed

**Figure 1:**
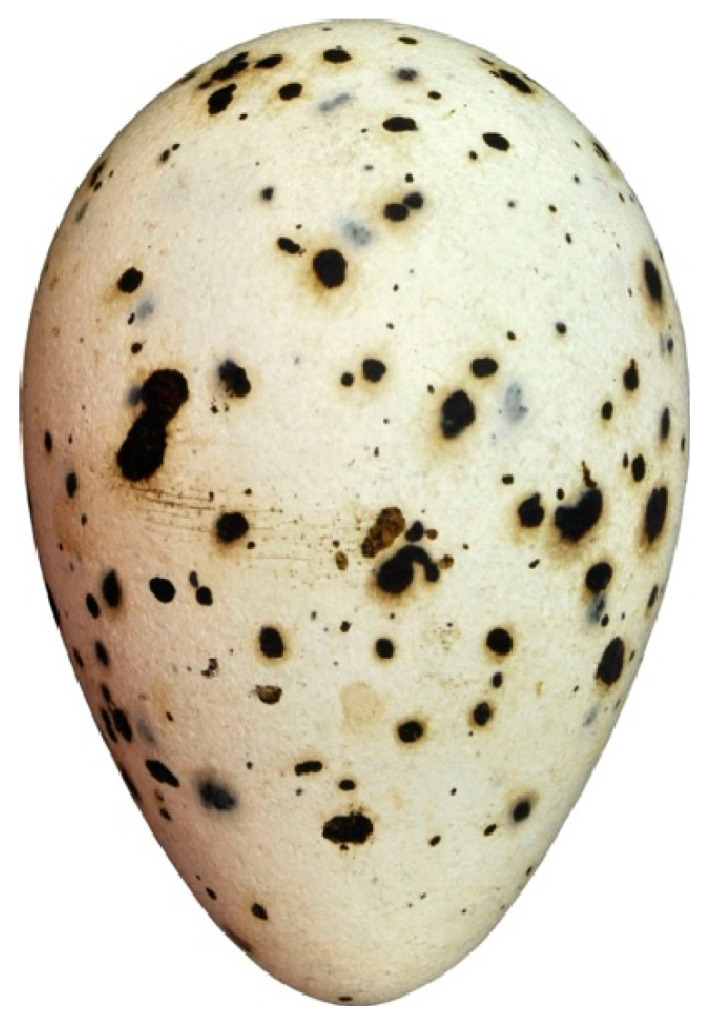
A pyriform egg of the royal tern (*Thalasseus maximus*). (Image source: https://commons.wikimedia.org/wiki/File:Thalasseus_maximus_MWNH_0387.JPG (accessed on 17 November 2024; by Klaus Rassinger and Gerhard Cammerer, 2012; Category: Eggs of the Natural History Collections of the Museum Wiesbaden; CC-BY-SA-3.0)).

The above examples demonstrate that, at the moment, the predominant applications of DL in egg poultry farming are in the recognition and analysis of images of both whole eggs and their individual regions. Such applications are useful in the non-destructive selection of eggs [52,53,54]. In order to be totally effective, however, it is essential that the entire DL procedure is well trained and is geared towards achieving the maximum return for the task at hand. Many of us, in many walks of life, have asked questions of computers only to receive answers that have little relation to the original meaning of our enquiry. By way of a frivolous example to illustrate this point, we used as a starting point a common term in egg-related research: “pyriform”. Pyriform literally means “pear-shaped” and applies to a non-standard shape of eggs not easily described mathematically and typically laid by some sea birds such as guillemots (e.g., [55,56,57]). Figure 1 shows a typical pyriform egg, that of a royal tern.

**Figure 2:**
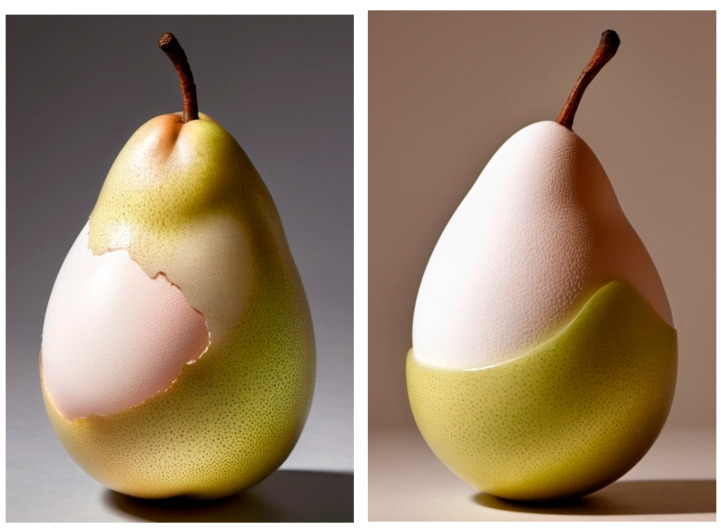
AI-generated images of pear-shaped eggs using OpenArt [58].

As scientists, we might expect that an AI program would perceive “pyriform egg” as something similar to Figure 1; however, by inputting the term into the OpenArt AI system [58,59], a strange “pear–egg hybrid” image was produced (Figure 2).

The point here is that the AI system was not properly trained to our needs and succeeded only in producing an amusing piece of surrealist art. Thus, a thorough, systematic and well-founded approach to training a system that uses AI, ML, NN and/or DL is necessary in order for it to be useful. This applies to all practical applications of AI, and particularly, in the context of this manuscript, industrial poultry farming and the implementation of research programs for use on intact bird eggs [60,61,62]. As with all computer systems, the “rubbish in, rubbish out” principle applies. With this in mind, the purpose of this review was to choose and analyze non-invasive, mathematical, egg-related studies, models and algorithms that will help in the process of DL using corresponding executive computerized systems to direct their main efforts to particularly prospective areas that are within the power of the current level of development of AI and NNs.

## 3. What Information Can Be Gleaned from Egg Shape?

### 3.1. Geometry of Egg Profile

In the last few years, works aimed at mathematically describing the geometry of a bird’s egg have increased dramatically after, ironically, a relative lull lasting more than half a century since the publication of pioneering works in this field [63,64,65]. One possible explanation is that the active use of AI and computational methods in many different walks of life, from ChatGPT to OpenArt and beyond, have reignited interest in this area, making it more accessible to a greater number of scientists. Almost simultaneously, several research groups began to work in the field of the development of and/or improvement in mathematical models of egg contours.

#### 3.1.1. Preston–Biggins Egg Model

A group predominantly from the University of Sheffield [66,67] took, as a basis, the Preston model [63], which is a product of two functions: the equation of a circle and a polynomial of the first, second or third order that can be used depending on the complexity level of the shape of a particular egg. These authors [67] demonstrated the accuracy of this model by using it in practice to describe a large number of actual contours of bird eggs, while developing a mechanism for initial measurements and refining the mathematical formula itself, which took the following form:

(1) $$\begin{array}{l} y=\frac{1}{24}\left[\left(-\frac{3}{\sqrt{7}}(d_{1}+d_{4})+\frac{27}{\sqrt{15}}(d_{2}+d_{3})\right)\right.\\ +\left(\frac{4}{\sqrt{7}}(d_{1}-d_{4})-\frac{108}{\sqrt{15}}(d_{2}-d_{3})\right)x\\ +\left(\frac{48}{\sqrt{7}}(d_{1}+d_{4})-\frac{48}{\sqrt{15}}(d_{2}+d_{3})\right)x^{2}\\ +\left.\left(-\frac{64}{\sqrt{7}}(d_{1}-d_{4})+\frac{192}{\sqrt{15}}(d_{2}-d_{3})\right)x^{3}\right]\sqrt{1-x^{2}} \end{array}$$

To use the proposed model (Equation (1)), it is necessary to measure the length of the egg, *L*, and the diameters, *d*_1_ to *d*_4_, at the points corresponding to ⅛*L*, ⅜*L*, ⅝*L* and ⅞*L*. Since the authors [67] developed the model in such a way that the formula is for the egg radius *y* at *x*, where −1 ≤ *x* ≤ 1 and the points of the egg are at *x* = −1 and *x* = 1, to find the values *d*_1_ … *d*_4_ in Equation (1), the measured diameters at the corresponding points should be divided by *L*. Thus, the resulting equation (Equation (1)), which can be called the Preston–Biggins model, without in any way diminishing the merits of the other authors of the team [67], can be attributed to the five-parameter egg formula.

#### 3.1.2. Hügelschäffer’s Model

In a similar way to the fact that that Biggins et al. [66,67] gave a second life to the Preston model [63], researchers from the University of Belgrade, Serbia, through extensive studies [68,69,70,71], revived Hügelschäffer’s model, as originally described in [72,73], and applied it to egg-shaped architectural structures. Narushin et al. [74] further modified Hügelschäffer’s model’s formula, adapting it to the parameters of a bird’s egg:

(2) $$y=\pm \frac{B}{2}\sqrt{\frac{L^{2}-4x^{2}}{L^{2}+8wx+4w^{2}}},$$

where *B* represents the maximum breadth of the egg, *L* represents its length and *w* is the parameter that displays the distance between two vertical lines that conform to *B* and ½*L*.

**Figure 3:**
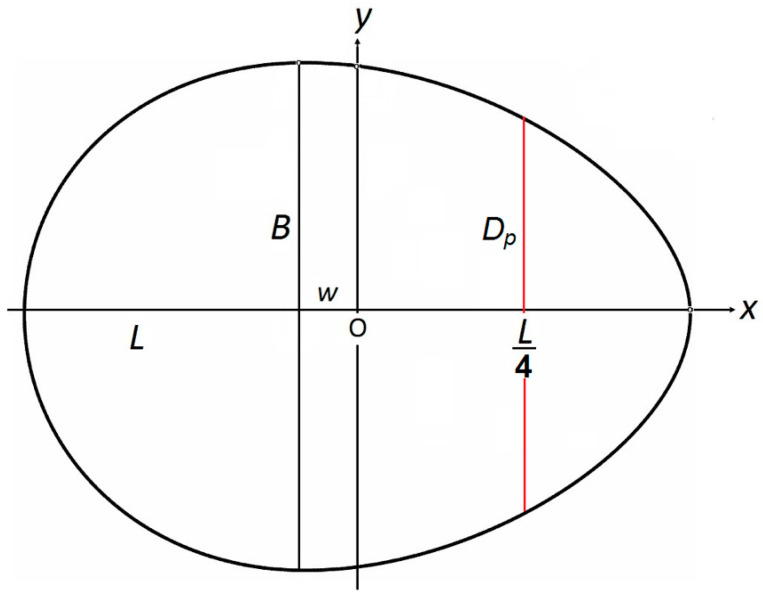
The three parameters (*B*, *L* and *w*) defining Hügelschäffer’s model (Equation (2)) for the description of an egg’s contours.

Figure 3 helps visualize the measured parameters in Equation (2).

In accordance with the number of parameters, Hügelschäffer’s model is a three-parameter model, which greatly simplifies the process of initial measurement. However, this equation (Equation (2)) can only accurately describe a limited set of the entire variety of bird egg shapes [55]. For example, the pyriform contours discussed above (Figure 1) turned out to be beyond its control. As a result of mathematical transformations, Hügelschäffer’s model was somewhat improved further in such a way as to be able to obtain a geometric image of eggs of exactly this shape [55]:

(3) $$y=\pm \frac{B}{2}\cdot \sqrt{\frac{(L^{2}-4x^{2})L}{2(L-2w)x^{2}+(L^{2}+8Lw-4w^{2})x+2Lw^{2}+L^{2}w+L^{3}}}.$$

The number of initial parameters required for the mathematical description of egg contours is of considerable importance both for manual and machine measurements. Because of this, a number of efforts were aimed at minimizing the number of indicators included in the mathematical model. In this regard, two two-parameter models were derived that can be used to describe the geometry of typical oval eggs characteristic of most domestic species [75]:

(4) $$y=\pm B\sqrt{\frac{x(L-x)}{L^{2}+4\left(0.103+0.012\frac{B}{L}-0.115{\left(\frac{B}{L}\right)}^{2}\right)(L-x)\left(L-2x\right)+4{\left(0.103+0.012\frac{B}{L}-0.115{\left(\frac{B}{L}\right)}^{2}\right)}^{2}{\left(L-x\right)}^{2}}}$$

and that of pyriform eggs [57]:

(5) $$y=\pm B\sqrt{\frac{Lx(L-x)}{2x^{2}\left(L-\left(1.212\sqrt{1-0.325{\left(\frac{B}{L}\right)}^{2}}-1\right)\sqrt{L^{2}-x^{2}}\right)+\left(L+\left(1.212\sqrt{1-0.325{\left(\frac{B}{L}\right)}^{2}}-1\right)\sqrt{L^{2}-x^{2}}\right)\left(\left(\left(1.212\sqrt{1-0.325{\left(\frac{B}{L}\right)}^{2}}-1\right)\sqrt{L^{2}-x^{2}}-3L\right)x+2L^{2}\right)}}$$

For each model, these are enough to measure only two basic geometric dimensions, i.e., *B* and *L*.

### 3.2. Universal Egg Models

The major problem that arises when creating a universal model to describe all geometric shapes of eggs is that it needs to apply to all eggs in nature, from spherical to pyriform. As a result of the mathematical transformations performed, Narushin et al. [55] proposed, in addition to *B*, *L* and *w*, to use an additional parameter, *D_p_*, i.e., the diameter of the egg at a point shifted from the pointed end by a distance of ¼*L* (shown in red in Figure 3), and created the following corresponding formula:

(6) $$y=\pm \frac{B}{2}\sqrt{\frac{L^{2}-4x^{2}}{L^{2}+8wx+4w^{2}}}\cdot \left(1-\frac{\sqrt{5.5L^{2}+11Lw+4w^{2}}\cdot (\sqrt{3}BL-2D_{p}\sqrt{L^{2}+2wL+4w^{2}})}{\sqrt{3}BL(\sqrt{5.5L^{2}+11Lw+4w^{2}}-2\sqrt{L^{2}+2wL+4w^{2}})}\cdot \right.\left.\left(1-\sqrt{\frac{L(L^{2}+8wx+4w^{2})}{2(L-2w)x^{2}+(L^{2}+8Lw-4w^{2})x+2Lw^{2}+L^{2}w+L^{3}}}\right)\right)$$

By this time, there were several other equations [76,77,78] that also claimed to be universal, that is, capable of describing the geometric contours of any egg. Biggins et al. [67] took on the question of which model was the most adequate, demonstrating that (i) the Preston–Biggins model (Equation (1)) was the most accurate in duplicating actual egg contours, and (ii) a number of models [64,76,77] could be considered derivatives of the Preston model, which should be recognized as universal.

The egg model proposed by Shi et al. [78] was based on the so-called Gielis superformula [79] and described the main egg types quite accurately. However, the practical use of the model required many measurements, based on which the equation coefficients are determined empirically. A more in-depth analysis of the Shi et al. [78] model was constrained by its parametric form.

Thus, the question of the principles of egg model universality logically arose. Is the criterion of the accuracy of copying the actual egg the only one?

### 3.3. Principles of Egg Universalism and Main Axiom

Narushin et al. [56] proposed a slightly different approach to defining both the universality criterion and the geometric standard of the ovoid. Firstly, these authors [56] proposed a geometric criterion that mathematical models describing egg shapes should conform to. This criterion was called the *Main Axiom of the Mathematical Formula of the Bird’s Egg* (hereinafter the *Main Axiom*). Essentially, this axiom states that the extremum of the function describing the contour geometry of the egg should be equal to *B*/2, or half the egg’s maximal diameter. Failure to comply with the Main Axiom principles leads to a violation of the basic dimensions of the egg in the model, most often the maximum breadth, *B*, and/or the magnitude of its shift from the center of the egg, *w*. Such changes in *B* and *w* result in a violation of the geometric principles. In this regard, this approach can become the main one in assessing the adequacy and compliance with the principles of universality and standardization of any mathematical model of the geometric profile of a bird’s egg. Secondly, Narushin et al. [56] proposed a mathematical model that corresponds to the Main Axiom and describes quite accurately the entire range of shapes inherent in bird eggs. Like the previous formula from these authors (Equation (6)), this one was also based on the measurement of four parameters, *B*, *L*, *w* and *D_p_*, as follows:

(7) $$y=\pm \frac{B}{2}\left[\frac{1}{\sqrt{1+4\frac{{\left(x+w\right)}^{2}}{L^{2}-4x^{2}}}}+\left(\sqrt{\frac{1+4\frac{{\left(w-w_{p}\right)}^{2}}{L^{2}-4w^{2}}}{1+4\frac{{\left(x+w_{p}\right)}^{2}}{L^{2}-4x^{2}}}-\frac{1}{\sqrt{1+4\frac{{\left(x+w\right)}^{2}}{L^{2}-4x^{2}}}}}\right)\left(1+\frac{x+w}{\sqrt{{\left(x+w\right)}^{2}}}\right)\left(\frac{x}{L}+\frac{1}{4}\right)\right].$$

The following formula is the calculation for the variable *w_p_* in Equation (7):

(8) $$w_{p}=L\left(\frac{{\left(\frac{D_{p}}{B}\right)}^{2}\left(1-4{\left(\frac{w}{L}\right)}^{2}\right)+3\frac{w}{L}}{3-4{\left(\frac{D_{p}}{B}\right)}^{2}\left(1-4{\left(\frac{w}{L}\right)}^{2}\right)}-\sqrt{{\left(\frac{{\left(\frac{D_{p}}{B}\right)}^{2}\left(1-4{\left(\frac{w}{L}\right)}^{2}\right)+3\frac{w}{L}}{3-4{\left(\frac{D_{p}}{B}\right)}^{2}\left(1-4{\left(\frac{w}{L}\right)}^{2}\right)}\right)}^{2}-\frac{1}{4}}\right).$$

Mathematical analysis of the Preston–Biggins model (Equation (1)) showed that this equation does not meet the requirements of the Main Axiom. Therefore, despite the accuracy of reproducing the contours of actual eggs, it cannot serve as a geometric standard.

### 3.4. Making Smart Smarter

It would seem that Equation (7) is the only one in the realm of egg models that satisfies the Main Axiom requirements. However, there is another basic model, which, after its author, can be called the Smart model [65,80,81]:

(9) $$\frac{x^{2}}{a^{2}}+\frac{y^{2}}{{\left(b+x\tan\theta \right)}^{2}}=1,$$

where *a* and *b* are the lengths of the semi-major and semi-minor axes of the ellipse, and *θ* is the angle of inclination of the tangential line to the horizontal at the midpoint of the surface of the formed egg.

In its presented form (Equation (9)), it is quite difficult to give an unambiguous estimate of the parameter tan*θ*, which is included in the Smart model. Narushin et al. [82] investigated a few possible options for determining this angle and came to the conclusion that in the case of following the Main Axiom principles, tan*θ* can be expressed through the basic geometric parameters of the egg, and the Smart model, as a result, can be reduced to the following form:

(10) $$y=\pm \frac{B}{2}\left(1+\frac{4w}{L^{2}-4w^{2}}(x-w)\right)\sqrt{\frac{L^{2}-4x^{2}}{L^{2}-4w^{2}}}.$$

The obtained dependence did not provide the highest accuracy for copying the profiles of actual eggs, but it did have a number of advantages. The main one is its compliance with the Main Axiom. Of no small importance is the fairly simple form and measurement of only three initial parameters of the egg, i.e., *L*, *B* and *w*.

### 3.5. Improving Baker

A similar geometric standard of the egg was obtained using the model derived by Baker [76] based on a fundamental transformation from projective geometry. The basic form of the Baker model for the upper half of the egg profile is as follows:

(11) $$y=Τ{\left(1+x\right)}^{\frac{1}{1+\lambda }}{\left(1-x\right)}^{\frac{\lambda }{1+\lambda }}.$$

Baker [76] explained the coefficients *Τ* and *λ* in Equation (11) as follows: *T* is an egg’s reciprocal equatorial elongation, and *λ* is a measure of the egg’s *asymmetry*. The difficulty in the practical use of the Baker model lies in the need to measure eight parameters, seven radii of the egg at different points and its length, as well as the fact that Baker [76], in his model, used not the actual dimensions of the egg, but those reduced to a conditional length equal to 2 (*x* = −1 … 1). In this respect, Narushin et al. [83] modified its mathematical form so that it included the parameters of the egg examined, which have already become typical for this type of research, and conformed to the Main Axiom principles:

(12) $$y=\pm Τ{\left(\frac{L}{2}+x\right)}^{\frac{1}{1+\lambda }}{\left(\frac{L}{2}-x\right)}^{\frac{\lambda }{1+\lambda }}.$$

Hereby, the parameters *λ* and *T* are determined by the following formulae:

(13) $$\lambda =\frac{L-2w}{L+2w},$$

(14) $$T=\frac{B}{2}\cdot \frac{1}{{\left(\frac{L}{2}+w\right)}^{\frac{1}{1+\lambda }}{\left(\frac{L}{2}-w\right)}^{\frac{\lambda }{1+\lambda }}}.$$

### 3.6. How “Main” Is the Main Axiom?

What is the reason for the necessity of following the Main Axiom when developing mathematical models describing egg shapes, and why is this fact so persistently emphasized in the studies by Narushin et al. [56,82]? The importance of such compliance lies in the basic principles of following any mathematical model to the postulates of classical laws, as can be outlined below:

- *Maintaining accuracy and predictability*. The geometry laws developed in ancient times have been tested in practice and provide accurate and predictable results. Compliance with these laws helps avoid errors and misunderstandings when solving problems, constructing theories and performing calculations.
- *Universality and consistency*. Classical geometric laws serve as the basis for many areas of mathematics and other disciplines, such as physics, engineering and architecture. A model that complies with these laws can be easily used in various fields where consistency with other mathematical and scientific concepts is important.
- *Practical application*. Geometry is widely used in real life—from the construction of buildings to drawing to technology development. Non-compliance with classical laws can lead to practical errors that can affect the strength of structures, the reliability of mechanisms and the accuracy of measurements.
- *Comprehension and interpretation*. Classical geometry laws are understood and familiar to a wide range of specialists, which facilitates the transfer of information, interpretation and comprehension. An inappropriate model would require additional training, e.g., in DL trials, and could cause confusion.
- *Unification of mathematical knowledge*. Classical geometry represents the fundamental knowledge that underlies more complex mathematical structures and theories. If a model violates these laws, it can affect further calculations and lead to errors in more complex mathematical theories.

Thus, compliance with classical geometry laws allows the maintenance of the accuracy, ease of use, interpretation and overall consistency of scientific and engineering solutions. Indeed, various mathematical models of eggs have found their most extensive and profound implementations in the sphere of engineering. These are, for instance, applications in the fields of thin-walled vessels and reservoirs [84,85,86,87,88,89], architectural structures [69,90,91,92], egg-shape lunar habitat structures [93,94], tsunami shelters [95], space structures modeling around black holes [96,97], telescopes [98] and a number of other, perhaps less scientific but no less common, areas of use, for example, computer games [99], artworks [100,101] and others.

## 4. AI and Egg Profiling

From the above, a natural question arises, namely, where exactly can AI be used in relation to the issue of the mathematical description of egg shape? This could pertain to both DL and ML, i.e., operations related to calculations using developed models.

Perhaps the most significant is when an object in nature shows a deviation from the geometric profile; in fact, this is relatively common in poultry eggs. In table eggs, for instance, a slightly “odd-shaped” egg can elicit a very negative reaction in a consumer in the same way as, for instance, a misshapen vegetable. In addition, the choice of eggs intended for incubation is quite scrupulous; specifically, it concerns the ratio of the breadth and length of the egg (*B*/*L*), known as the *shape index*. Such a criterion is extremely informative for the egg industry, and attempts to use it for ML have already been made [13]. We discuss the relationships (or indices) between various egg parameters in more detail in Section 5.

As with many biological studies, deviations from the norm (in terms of DL studies of bird eggs) are performed by comparison to an agreed standard for a specific species, e.g., chickens. This approach was proposed by Narushin et al. [55,56]. According to this premise, each egg has its own standard profile, which can be reproduced by measuring four parameters, i.e., *L*, *B*, *w* and *D_p_*, and entering them into Equations (7) or (10). By superimposing an image of the standard profile on the actual one for, say, eggs with a successfully hatched embryo and a dead one, it is possible to teach AI which shape deviations can lead to negative consequences. In a laboratory or industrial setting, machine vision needs to be significantly superior to the subjective judgment of the human eye. Indeed, it can be made to be so through the application of a huge number of measurements.

The relationship with the shape is not only possible for identifying hatching waste (i.e., the proportion of eggs that do not hatch). There is a high probability that the geometric features of an egg’s contours can even indicate the possible sex of its embryo. In any case, a number of studies have shown a relationship between sex and the *B*/*L* ratio (e.g., [102,103]). Although such a relationship did not always demonstrate significant results (e.g., [104]), the simplicity of measuring the shape index quite often prompted researchers to use it to solve one of the most important problems in modern poultry farming, i.e., pre-incubation sex detection [105,106,107,108,109]. Perhaps analyzing a larger number of geometric parameters or the degree of conformity of the egg contours with their standard will help to bring the solution of this egg sexing problem even closer. The only thing to emphasize is that the use of AI and its training algorithm is not a guarantee for solving all of the technological problems pertaining to egg industry. Firstly, large volumes of high-quality data are required to train AI. A lack of data or their poor quality can reduce the efficiency of models. Therefore, a lot of further work is required to obtain high-quality data and fill DL models with information. Secondly, and perhaps most importantly, there is the matter of the repeatability of results. A model can remember the training sample, but will poorly generalize new data if it does not perfectly duplicate those on which training took place. In this case, its practical value is reduced.

Another issue of concern is the multitude of mathematical models describing the geometry of egg contours and which of them are applicable to which specific purpose. Some will be useful in particular applications, while others will be better applied in quite different situations. We have attempted to answer this question in the form of the following postulates aimed at the legitimacy of the existence, development and practical application of various mathematical models to describe the same object:

- *Complex comprehension*. Each model focuses on different aspects of the object, allowing for a more complete and multifaceted understanding of its properties.
- *Validation*. Different models allow one to compare results and identify potential errors. If several models give similar results, this increases confidence that they correctly describe the object.
- *Adaptability*. Different models may be useful in different conditions and for different purposes. For example, one model may work better for quick calculations, another for analysis over a longer period of time and a third for accurately accounting for individual nuances of the object being described.
- *Overcoming limitations*. Each model has its limitations and simplifications. By using several models, one can compensate for the weaknesses of one by using the strengths of another. This is especially important if the object is difficult to describe with one model without significant assumptions.
- *Possibility of prediction and optimization*. Different models can allow different predictions to be made about the behavior of an object when parameters change. This can help in the search for the optimal conditions or methods of controlling the object depending on the goals set.

Collectively, we can conclude that the combination of different models not only allows one to understand the object itself more deeply, but also to use the mathematical description in applied problems more accurately, adaptively and confidently. Thus, both the usage of existing models and the development of new ones can be welcomed. In other words, different models have their pros and cons, and using them in combination may well yield more useful results than relying on single models alone.

## 5. Power of Indices

### 5.1. B/L, or Classical Shape Index

The aforementioned egg shape index (*B*/*L*) is arguably the oldest and most popular index used as a geometric characteristic of an intact egg. According to Hamilton [110], who devoted an entire section of his work “*Who published it first?*” to the issue of restoring historical and scientific justice regarding the terminology proposed for eggs, the founders of the shape index were Dunn and Schneider [111].

Research on the relationship between the incubation properties of eggs depending on the *B*/*L* value began, perhaps, from the moment this index was published. It would seem that the results of the published datasets are quite sufficient for making practical decisions on the issue of the pre-incubation sorting of eggs. However, more and more new studies on this topic, for both poultry and wild species, continue to find their readers [13,112,113,114,115,116,117,118].

### 5.2. Other Indices

In addition to the shape index (*B*/*L*), many others have been developed that characterize the geometric features of egg contours. The most logical and simple ones have been known since the publication of Preston [119,120]: asymmetry, or the extent to which one end is larger than the other, and *bicone*, determined based on measurements of the radius of curvature at the blunt end (*R_B_*) and at the pointed end (*R_P_*). In this case, Preston [119,120] used the inverse ratio of the length and breadth of the egg, *L*/*B*, calling it the *index of elongation*.

Over the course of subsequent research, geometric indices were supplemented, for example, by the *conicity index* [121,122,123], or *pointedness* [77]. The calculation formulae for their determination were further transformed or slightly modified [124,125,126].

However, these indices were only used to describe the shape of eggs, i.e., they served as an alternative to mathematical models. Their practical use is only appropriate if there is a correlation with, for example, the success of incubation and/or other properties that carry some economic sense. Nevertheless, such results for effective implementation in commercial poultry farming are extremely rare. Beginning with the first studies on the search for possible relationships between egg shape and hatchability (e.g., [127]), their authors cautiously stated that “*narrow eggs rarely hatch*”, while avoiding any technical details with the help of which it would be possible to describe the concept of “narrow eggs” using mathematical indices [127]. Judging by the analysis of similar publications, researchers who have studied the relationship between egg shape and incubation results continue to limit themselves to using abstract characteristics of “narrow eggs” and “round eggs” instead of clear mathematical values.

The use of the geometric indices of wild species eggs, however, has given impetus to a number of evolutionary hypotheses in ornithology. Montgomerie et al. [126] studied the egg shape in 955 extant bird species and concluded that the elongation ratio (*L*/*B*) was influenced by the constraints imposed by the female’s anatomy during egg formation. On the other hand, egg asymmetry was mainly associated with clutch size and nesting features, i.e., factors that affect the thermal efficiency of the incubation process and the risk of shell damage. Herewith, to assess asymmetry, Montgomerie et al. [126] combined the asymmetry and bicone indices, renaming them *pointedness* (*P*) and *polar asymmetry* (*A*), and also changing the formulae for their calculation, in comparison with those proposed by Preston [119,120]. Based on the notations adopted by us in this article, presented in Figure 3, the *P* value is determined as follows:

(15) $$P=\frac{\frac{L}{2}+w}{L}=\frac{1}{2}+\frac{w}{L}.$$

The *A* value, according to Montgomerie et al. [126], was either defined as the ratio of the diameters on the side of the blunt and pointed ends, or, if applying the notation of Preston [119,120], used the corresponding radii:

(16) $$A=\frac{R_{b}}{R_{p}}.$$

According to the studies by Stoddard et al. [128,129], egg shape is directly related to the ability of birds to fly. That is, the adaptation mechanisms for flight could be critical factors in the variability of egg shape. In order to maintain a streamlined body shape necessary for flight, birds lay more asymmetrical or elliptical eggs. Accordingly, these authors [128] used asymmetry and *ellipticity* indices when analyzing the results of their studies based on the parameters included in the Baker model (Equation (11)), i.e., the value of *λ* − 1 as a measure of the asymmetry of an egg and *T* as an indicator of ellipticity. A simpler mathematical transformation of Formulas (13) and (14) derived by Narushin et al. [83] for a modified form of the Baker model (Equation (12)), which consists of dividing the numerator and denominator by *L*, shows that the parameter *λ* is nothing more than a function of the ratio *w*/*L*, and *T* is that for the set of indices *B*/*L* and *w*/*L*.

### 5.3. Three Pillars Among Indices: B/L, w/L and D_p_/B

The variety of indices used by various authors in their studies, their different names and their approaches to computation require some unification. The work of Biggins et al. [66] can perhaps be considered the first in which the authors provided a list of the most commonly used indices and attempted to conduct a comparative analysis of them.

Based on numerous theoretical and practical studies, Narushin et al. [130] concluded that the entire variety of geometric indices could be reduced to three main ones: the shape index (*B*/*L*); the *displacement index* (*w*/*L*); and the conicity index (*D_p_*/*B*). In essence, the *w*/*L* index can serve as a characteristic of egg asymmetry, that is, it indicates the degree of egg shape deviation from the ellipse. Thus, the *w*/*L* ratio can equally be called the asymmetry index.

### 5.4. L/T Index and Emergency Geometrical Index

In some cases, indices that combine geometric parameters of both the external and internal structures of the egg prove to be quite effective. Narushin et al. [131] successfully used the ratio of *L* to the shell thickness, *T*, in their studies on the depth of the so-called neutral line of the shell. This is a certain imaginary line that has the same length after bending as it had before bending [132]. The use of the *L*/*T* index in interaction with others (*B*/*L* and *w*/*L*) contributed to the derivation of a calculation formula for determining the so-called *k-factor*, which is a ratio of the location of the neutral line to the shell thickness [131]. The k-factor value is directly related to the strength of the material under study [133,134,135,136,137]. In this regard, the *L*/*T* index may have practical value as an indicator of eggshell strength. In addition to strength, the *L*/*T* value turned out to be a good predictor of shell density [138].

The use of indices in a complex manner, rather than separately, may be a very promising solution. However, we are not talking about their parallel use. This may be the creation of separate indices calculated according to a certain mathematical expression that includes other indices. For example, Narushin et al. [139], studying the results of goose egg incubation, found that to predict the success of hatching, it is advisable to use such a complex set that the authors called the *Emergency Geometrical Index* (*EGI*). As a result of some mathematical transformations of the original formula proposed by Narushin et al. [139], the *EGI* value is influenced by the shape and conicity indices:

(17) $$EGI=\frac{1}{{\left(\frac{B}{L}\right)}^{2}\cdot \frac{D_{p}}{B}}.$$

The authors [139] concluded that with a certain degree of certainty, it can be stated that goose eggs with high *EGI* values have every reason to have increased embryo mortality during their incubation.

### 5.5. Index Importance, Variability and DL Implications

**Figure 4:**
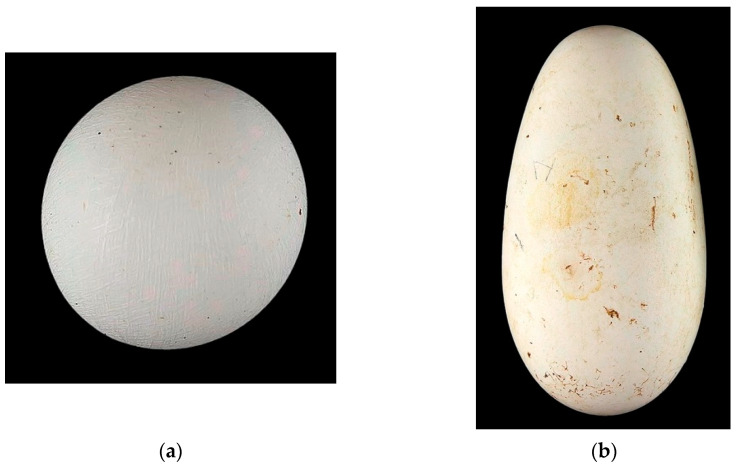
Examples of spherical (**a**) and elongated (**b**) avian eggs. (**a**) Pin-tailed whydah (*Vidua macroura*); https://commons.wikimedia.org/wiki/File:Vidua_macroura_MHNT.Z.2010.11.150.20.jpg. (**b**) African sacred ibis (*Threskiornis aethiopicus*); https://commons.wikimedia.org/wiki/File:Threskiornis_aethiopicus_MHNT.ZOO.2010.11.63.1.jpg (accessed on 17 November 2024; by Roger Culos, collection of Jacques Perrin de Brichambaut; CC-BY-SA-4.0).

Why are such indices so important? They allow us to narrow down the set of parameters and their variability, having a significantly smaller number of variability options. Using the shape index as an example, if we consider the variability options *B* and *L* for the entire variety of bird eggs among the existing species, their values can vary from 125 × 167 mm for ostriches (e.g., [140]) to 8 × 12 mm for a hummingbird egg (e.g., [141]). If we operate with the ratio of these values, the *B*/*L* variability is within 0.5 … 1.0 (e.g., [55,130]), which gives significantly fewer options for egg gradation. Thus, by selecting images of eggs whose *B*/*L* values are within a certain interval of our interest, we can train AI to select them according to this criterion, regardless of the values of the actual sizes of *B* and *L*. Similarly, the DL process can be carried out for any other indices. In this case, the question arises about their degree of possible variation. For *B*/*L*, this is quite simple, and within the entire diversity of the bird kingdom, one can find eggs with a shape index of both 0.5 and 1.0. A visual illustration was presented in the work by Narushin et al. [130], which we will also provide in this review (Figure 4).

As for the *w*/*L* index, the possible variability of its values was determined theoretically [55] from 0 to 0.25. However, later studies [56,57], aimed at studying eggs with high asymmetry values, made it possible to detect real images of eggs in which the maximum *w*/*L* value reached only 0.14. Thus, in their further studies [130], these authors propose to take into account the variability limits of *w*/*L* = 0 … 0.2.

Also, Narushin et al. [130], based on the theoretical research conducted, proposed an approach to determine the degree of variability of the conicity index (*D_p_*/*B*). Its lower limit (minimum), inherent in pear-shaped eggs, conforms to the parabolic shape [55,56,57] and is determined by the previously derived formula [142] as follows:

(18) $${\frac{D_{p}}{B}}_{\min}=\frac{0.707}{\sqrt{1+2\frac{w}{L}}}.$$

Its upper value (maximum) is limited by the ovoid shape, as determined by the Hügelschäffer’s model [74,142]; is characteristic of, for example, chicken eggs; and can be defined by the following dependence:

(19) $${\frac{D_{p}}{B}}_{\max}=\frac{0.866}{\sqrt{1+2\frac{w}{L}+4{\left(\frac{w}{L}\right)}^{2}}}.$$

Thus, the conicity index value is directly related to the value of the asymmetry (displacement) index (*w*/*L*) by strict mathematical dependencies.

## 6. Egg Volume and Surface Area in Detail

### 6.1. How to Compute V and S

Mathematical calculations related to bird eggs have gained the greatest popularity in deriving formulae for determining their volume, *V*, and surface area, *S*. The relevance of these indicators is extremely difficult to overestimate, since they provide a lot of useful information for both research and practical purposes. Machine vision systems efficiently apply computational mathematical models to sort a continuous flow of table eggs in an industrial setting (e.g., [64,143,144,145,146,147,148]). There is also a huge variety of computation algorithms for which, thereby, the authors declare a fairly high accuracy after performing an appropriate experimental verification. There is no point in listing them all, since more and more new formulae appear; although, it would seem, this area has been thoroughly studied. In this regard, we decided to focus in this review on (i) the main errors that are often encountered in research practice, and (ii) on recommendations for simplifying the derivation of the respective computation equations for a specific population or species of birds examined.

Often, for practical purposes, it is not required to determine *V* and *S* accurately enough. However, the use of a limited set of initial parameters is quite relevant. Researchers take as a basis two initial calculation formulae that, based on older, nevertheless pioneering publications at that time [149,150,151,152,153], were later conditionally presented (e.g., [154]) in the following form:

(20) $$V=k_{V}LB^{2},$$

(21) $$S=k_{S}V^{\frac{2}{3}},$$

where *k_V_* and *k_S_* are hypothetical constants.

As a result, the experimenter’s task is reduced to the use of a direct measurement procedure (e.g., Archimedes’ principle) to determine the *V* of a certain sampling of eggs, measure their length (*L*) and maximum diameter (*B*), and substitute these data into Equation (20). The final recalculation will give the desired average coefficient *k_V_*. Naturally, the spread in *k_V_* values will affect the accuracy, as will eggs with sizes *L* and *B*, the values of which are outside the experimental sampling. There is an indisputable truth that “*prediction equations derived from one data set are generally not transferrable to another*” [110]. However, for most applied problems, the above calculation algorithm will be quite sufficient.

Calculating *S* is much more complicated. After all, there are no direct accurate methods for measuring this value on an egg. As an alternative to direct measurement, it is possible to use the 2D imaging method. However, its use also does not ensure sufficient accuracy (e.g., [142]). It is for such situations that developed mathematical formulae for the geometric profiles of eggs provide an indispensable aid. Classical formulae of integral geometry allow one to compute a lot of useful parameters for figures of revolution based on their relevant mathematical equation. These include the already-mentioned *V* and *S*, as well as the area of the normal/orthogonal projection of an egg, *A*, the long circumference length, *C*, and the radius of curvature, *R*, at any point on the egg.

### 6.2. Deeper Computations of V and S

Even in his first publication, the founder of the direction of the mathematical description of the geometry of egg profiles, Preston [63], wrote that this kind of research is “*not undertaken primarily as a mathematical amusement*”. Therefore, the development of each of the models was accompanied by the derivation of computation formulae for *V* using integral calculations. As a result, the appropriate dependencies were obtained for the Preston model [66,155], Baker model [76], and Smart model [156]. Unfortunately, these authors limited themselves to only deriving the respective formulae for calculating *V*. The integral calculation of *S* is quite complex, which, obviously, was the reason for the lack of mathematical dependencies to determine this parameter. However, a number of papers have appeared in which the authors did use a few alternative approaches to deduce dependencies for calculating the *S* value. We list these methods below.

The first and the simplest, although the least accurate, approach was to use simplified methods for computing complex integrals. For example, Narushin et al. [74] used Simpson’s rule along with the corresponding integration of Hügelschäffer’s model to derive a formula for determining *S*.

The second method is to dismember the model into several simpler parts and then integrate each of the parts separately. Herewith, complex integrals are defined as the sum of the areas of single trapezoids into which each of the parts is divided. This procedure was effectively applied by Narushin et al. [142] to derive calculation equations for *V* and *S* along with the appropriate integration of complex egg profile models [55,56]. As a result, the obtained equations based on the model corresponding to the Main Axiom [56] can be considered universal and suitable to find the *V* and *S* values of an egg of any shape as follows:

(22) $$V=\frac{\pi }{128}\left[\left(8.917-29.998\frac{w}{L}\right){\left(\frac{D_{p}}{B}\right)}^{2}+\left(2.459+88.647\frac{w}{L}\right)\frac{D_{p}}{B}-36.26\frac{w}{L}+12.453\right]LB^{2},$$

(23) $$S=\pi BL\left(0.389+0.188\frac{B}{L}-0.063\frac{w}{L}+0.365\frac{D_{p}}{B}+0.114\frac{D_{p}}{B}\cdot \frac{B}{L}-0.168\frac{w}{L}\cdot \frac{B}{L}+0.46\frac{w}{L}\cdot \frac{D_{p}}{B}+0.484\frac{w}{L}\cdot \frac{D_{p}}{B}\cdot \frac{B}{L}\right).$$

The third approach relies on the usage of a topological approach (e.g., [157]). According to this method, the egg is conditionally transformed into a certain standard geometric figure of the same volume, for which exact formulae for calculating *S* have already been derived. This approach was efficiently implemented in the studies by Narushin [121] and Narushin et al. [6], with a few versions of topological transformations also tested. An ellipsoid and Narushin’s model [121] were considered as a standard figure that was recognized as optimal for computing the *S* value based on the egg volume, *V*, measured by the direct Archimedes principle. The final calculation formula is as follows:

(24) $$S=\left[6.438-2.666\frac{B}{L}+1.867\frac{w}{L}-0.44\frac{D_{p}}{B}-0.134\frac{D_{p}}{B}\cdot \frac{B}{L}-0.683\frac{B}{L}\cdot \frac{w}{L}-2.578\frac{w}{L}\cdot \frac{D_{p}}{B}+1.29\frac{w}{L}\cdot \frac{D_{p}}{B}\cdot \frac{B}{L}+1.369{\left(\frac{B}{L}\right)}^{2}+0.336{\left(\frac{w}{L}\right)}^{2}+0.233{\left(\frac{D_{p}}{B}\right)}^{2}\right]V^{\frac{2}{3}}.$$

Knowing the possible variations in the parameters *L*, *B*, *w* and *D_p_* and/or their indices (*B*/*L*, *w*/*L* and *D_p_*/*B*), characteristic of eggs of a certain species or taxonomic group of birds, and having an accurate calculation formula for *V* and/or *S* at hand, one can complete the required computations without performing complex experiments at all. For this purpose, it is sufficient to either substitute the average values of the input parameters, for example, into Equations (22) or (23), or implement the simulation procedure when creating a database of parameters for some virtual eggs that can be used to approximate the resultant set to deduce empirical and simpler calculation formulae. A similar approach was taken by Narushin et al. [154] when deriving simplified equations for calculating the *V* and *S* values of chicken eggs.

The method for obtaining simplified equations turned out to be so straightforward and efficient that we decided to illustrate it with a specific example. Let us assume that it is necessary to develop a simple formula to compute *V* and *S* for eggs of the royal tern (*Thalasseus maximus*) (Figure 1). To calculate the key indices included in Equations (22) and (23), we do not even need to have data on the actual dimensions of the eggs. Pixel data are sufficient, which can be easily measured on the image of the eggs using, say, the Microsoft Picture Manager program. The measurements performed demonstrated the following results: *B*/*L* = 0.680, *w*/*L* = 0.116 and *D_p_*/*B* = 0.733. Substituting the indices into Equations (22) and (23) yielded the following results:

(25) $$V=0.503LB^{2},$$

(26) S=2.788BL.

Thus, by means of simple recalculations, it is possible to produce simplified formulae and determine the main characteristics of an egg (*V* and *S*); although, without a doubt, the implementation of appropriate measurements of all parameters (*L*, *B*, *w* and *D_p_*) and the use of Equations (22) and/or (23) would make it possible to warrant more accurate data.

### 6.3. Beauties of S/V Index and DL Prospects

What additional benefits can the knowledge of the *V* and *S* of an egg provide? The answer, again, is in the “magic” power of indices. As aforementioned, individual parameters, combined into a certain mathematical relationship, can synergistically enhance their informational component. Thus, if we consider the ratio of the two values, *S*/*V*, we can discover a surprising effect from using this index. In principle, the *S*/*V* ratio is nothing new in biological research. It is extremely important since it determines the efficiency of metabolism in the body. A higher *S*/*V* ratio means that there is a larger surface area available for metabolism. This makes it easier for the body to absorb the necessary nutrients and remove waste products. In this regard, the *S*/*V* value may be an indirect indicator of metabolism (e.g., [158,159,160,161]). Atanasov [162] was perhaps the first to suggest considering a possible relationship between *S* and *V* (although in his analysis, he used the inverse value, *V*/*S*) on the one hand, and the incubation duration (*I*) of bird eggs on the other. The correlation coefficient found by Atanasov [162] between these values was *R* = 0.561. It is possible that the reason for a lower correlation was an insufficiently accurate method for calculating the *V* and *S* values, the data for which were taken by Atanasov [162] from the work of Hoyt [153], and/or was due to the limited experimental sampling, which consisted of 28 eggs. Narushin et al. [163], in their similar analysis, used data from a sampling of 444 egg images from 444 avian species, 89 families and 30 orders. As a result, the authors confirmed the relationship between the *S*/*V* value and *I* at the level of *R* = −0.870. Hereby, Narushin et al. [163] proposed using an empirically derived formula for a possible recalculation of the *I* value as follows:

(27) $$I=32.638{\left(\frac{S}{V}\right)}^{-0.635},$$

with *R*^2^ = 0.757 (*p* < 0.05), where *I* is expressed in days, *V* in cm^3^ and *S* in cm^2^.

A similar relationship was confirmed in a study of pyriform eggs of the Alcidae family [130]. Another investigation into the causes of disproportionately short incubation periods of cuckoo eggs [164] allowed the hypothesis that there may be some contribution to the *I* value from the shell thickness (*T*) of the incubated egg as follows:

(28) $$I=33.83{\left(\frac{S}{V}\right)}^{-0.56}\cdot {\left(\frac{T}{S}\right)}^{-0.06},$$

with *R*^2^ = 0.727 (*p* < 0.05), in which *I* is measured in days, *S* in cm^2^, *V* in cm^3^ and *T* in μm.

If the *S*/*V* ratio can be called the *metabolism index* of the developing embryo, the *T*/*S* index reflects the degree of distribution of the shell layer along the egg surface. Both indices are indirect indicators of the duration of incubation of bird eggs. Hereby, their increase leads to a decrease in the incubation period [164].

These and the above-mentioned data demonstrated a number of prospective indices that include the *T* value. For their practical use, non-destructive measurement tools for this indicator are needed. Such tools have been developed as both industrial and experimental versions. There are commercially accessible tools for measuring shell thickness, e.g., the non-destructive deformation instrument from Stable Micro Systems [165] and the ultrasonic device from ORKA [166]. The *T* prediction accuracy using non-destructive deformation tools can be increased by changing the nature of shell loading [167,168,169,170]. Among laboratory equipment, terahertz time-domain spectroscopy can also be a very promising piece of equipment for *T* prediction [171].

How can the obtained computation formulae be employed for DL applications? First of all, this can be achieved through the formation and sorting of images by the value of the *S*/*V* ratio. After all, egg recognition with reference to this parameter promises considerable benefits in terms of, for instance, possible pre-incubation egg sorting in order to synchronize the hatching of chicks. Again, it is possible that the incubation regime may also depend on this indicator, the optimization of which, based on the *S*/*V* value, can contribute to increasing hatchability. In any case, AI trained to recognize such eggs will help manage the search for possible patterns if they really exist.

## 7. How Useful Is Air Cell Information?

### 7.1. Measuring Air Cell

**Figure 5:**
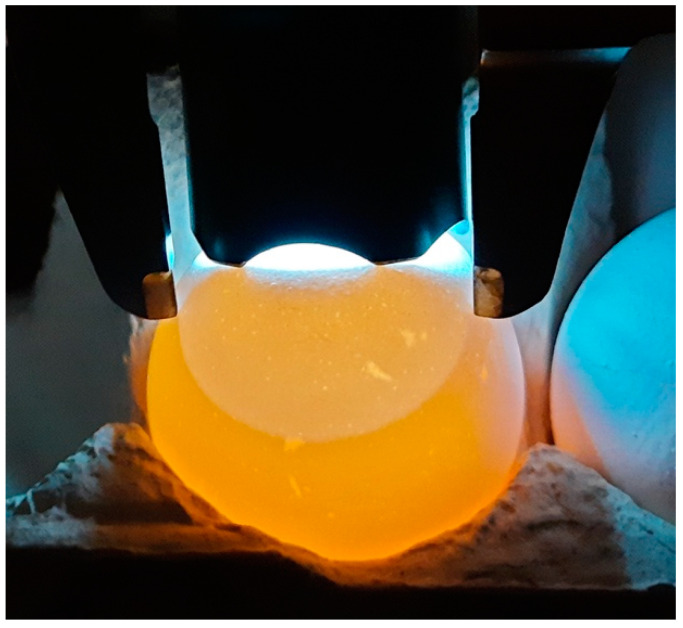
Measuring the diameter of the egg air cell using a caliper [173].

The air cell is a vital structure of the egg, providing the developing embryo with air during the activation of its pulmonary respiration. By increasing in size both during incubation and during possible storage time, the air cell can serve as a useful bioindicator for the processes of physiological change in the egg contents [172]. The air cell size can be easily recorded by simple measurement methods, as, for example, was demonstrated in the study by Narushin et al. [173] (Figure 5).

The measured air cell diameter, *d*, is easily converted into its height, *h*, and/or volume, *V_ac_*, using the calculation formulae proposed by Narushin et al. [174]:

(29) $$h=\frac{LB^{2}-2d^{2}w-\sqrt{{\left(2d^{2}w-LB^{2}\right)}^{2}-d^{2}B^{2}{\left(L-2w\right)}^{2}}}{2B^{2}},$$

(30) $$V_{ac}=\frac{1.32B^{2}h}{L^{3}}(h(L^{2}+3.79Lw-4.08w^{2})+0.012L(L^{2}-5.92Lw+172.58w^{2})),$$

where *h* is the air cell height (in cm), *d* is its diameter (in cm), and *V_ac_* is its volume (in cm^3^).

Machine technologies have been developed to automate the air cell measurement process. For instance, the ovoscope and the ultrasonographic imaging techniques used in the studies by Önler et al. [175] or tomographic images that allow the egg contents to be assessed [51] can be used to detect the air cell parameters. Automatic egg candling machines that can be used to determine, among other things, the air cell dimensions (e.g., [176]) are already actively employed in the industry.

### 7.2. Air Cell and DL Applications

Based on the existing DL developments aimed at analyzing the air cell dimensions [14,34], the technical side of generating the relevant images has been worked out. The question remains in the direction of AI training in order to obtain the maximum return on egg grading using the indicator of change (or prediction of possible changes) of the air cell parameters, or a set of indicators that include its dimensions. In this respect, a number of positive results have been reported that can be effectively involved in the DL process.

#### 7.2.1. Egg Storage

Firstly, these DL applications can be related to the process and terms of the possible storage of eggs before their further use as table eggs or for incubation. Exceeding these terms may lead to the fact that the egg may lose its nutritional or hatching properties during storage. Due to their individuality, there are no uniform terms that are the same for all eggs, even if they are laid on the same day from a homogeneous flock of laying hens. If for one group of eggs laid for preliminary storage, a two-week storage period may be quite acceptable, then for others it will be completely harmful. In this regard, there is the problem of a differential approach, dividing eggs by their possible potential of acceptable storage time. Narushin et al. [173] chose the egg shrinkage factor, i.e., the loss of mass (Δ*W*) during storage, as a criterion for changing the quality indicators of egg contents. As a result of 15-day storage, the Δ*W* value, defined as the difference between the initial egg weight and that at the end of the storage period, was approximated quite accurately using an empirical relationship. The parameters included in this relationship can be measured quite easily before placing eggs in storage. For this review, we decided to slightly modify the presentation of the obtained relationship in order to highlight the metabolism index that is an integral part of this formula:

(31) $$\Delta W=5.55W^{-0.29}\cdot {\left(\frac{S}{V}\right)}^{-5.22}\cdot d^{0.69},$$

with *R*^2^ = 0.847. 

The value of *W* is taken in g, *V* in cm^3^, *S* in cm^2^ and *d* in cm.

Thus, the DL process can be much more efficient if the eggs are pre-sorted by the metabolism index (*S/V*), after which the initial air cell size is estimated in each group. This approach will allow a decision to be made on the potential of each individual egg for its acceptable shrinkage and, accordingly, to assign an optimal shelf life for it.

#### 7.2.2. Egg Incubation

The second important area of the skillful use of air cell data may be the process of egg incubation itself.

**Figure 6:**
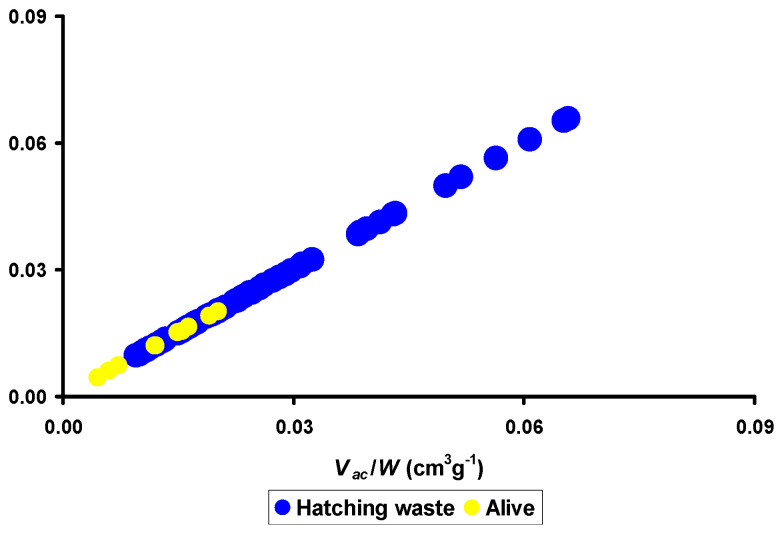
Visualization of the distribution of *V_ac_*/*W* values for the HW (blue dots) and A (yellow dots) egg groups in the general line of goose eggs examined [139].

In their study of the incubation potential of goose eggs, Narushin et al. [139] found that another index, representing the ratio of the air cell volume to the egg weight, *V_ac_*/*W*, allowed them to correctly identify 38% of all unfertilized eggs and 47% of non-viable embryos before they were put into the incubator. By combining both groups (unfertilized and non-viable) into a single one that was named “hatching waste” (HW), the authors [139] visualized the distribution of the eggs in this group in comparison with viable embryos, classified as “alive” (A) (Figure 6).

Thus, using the *V_ac_*/*W* indicator, it was possible to identify eggs with viable goose embryos in the zone of the small values of the general line of eggs studied (Figure 6). That is, the value of the *V_ac_*/*W* ratio can be taken as the basis for the DL process by training AI to recognize the size of the air cell among eggs of different weight categories.

However, Narushin et al. [139] proceeded further in their analysis of goose eggs, deciding to combine two indices, i.e., the above-mentioned *EGI* and the *V_ac_*/*W* ratio, which turned out to be quite promising. As a result of this index, “symbiosis”, a new indicator was born that was called the *Emergency Hatching Index* (*EHI*):

(32) $$EHI=\frac{W}{EGI\cdot V_{ac}}.$$

An analysis of the new index demonstrated that the viability of goose embryos increased with an increasing *EHI* value [139].

Although the task for the DL process has become more complicated, it has not become impossible. Most likely, it will be a question of combining several systems: the preliminary sorting of eggs by geometric and/or weight indicators, after which images of the corresponding graded eggs can be used for training on the size of the air chamber.

## 8. Three Key Parameters of an Egg

### 8.1. Weight, Volume and Surface Area

What is the most important parameter of an egg? The answer is obvious: it is its weight, *W*. The greater the weight of the egg itself, the heavier its components. In this regard, egg weight has long served as the main parameter for predicting both the structure of the egg and the physiological processes occurring in it. A team of scientists led by the American professor Hermann Rahn (1912–1990; see the respective biographical memoir by Pappenheimer [177]), based on the results of their own research as well as data on the morphological parameters of eggs from the oological reference book by Max Schönwetter [178], derived a large number of allometric relationships between various parameters of bird eggs and their weight, *W* [149,179,180,181]. It is quite obvious that if we are talking about the whole variety of bird eggs existing in nature and weighing approximately from 0.5 g to 1.5 kg, the relationship of *W* with the internal egg parameters is quite close among them. If we operate with eggs of one species group and, often, one industrial flock, it becomes more difficult to obtain an accurate dependence, although such attempts have also been made repeatedly. For example, Narushin [182] presented a few computation equations for an enlarged prediction of the weight of the shell, *W_s_*, yolk, *W_y_*, and albumen, *W_a_*, depending on the weight of the whole egg for some poultry species (chickens, turkeys and geese). Later, this list was supplemented with a prediction model for quail eggs [183]. Research aimed at the selection of individual structural egg components, improving incubation technology and analyzing hatchability and physiological characteristics of embryo development [184] requires more accurate prediction methods that would allow the calculation of the values of *W_s_*, *W_y_* and *W_a_* without destroying the egg. Such an attempt was made by Narushin [185], who found that the three main egg parameters, i.e., *W*, *V* and *S*, taken together, manage to solve this prediction problem much better than when taken separately, at least for chicken eggs. Later, Narushin et al. [186] demonstrated that using these same three parameters, it is possible to predict other variables of the chicken eggshell: thickness, density, fracture force and stiffness. This was also possible to achieve for the internal parameters of quail [6] and goose [187] eggs.

How can the parameters *W*, *V* and *S* be used for the relevant analysis of egg images in the DL process? After all, there are a great many variants of the various combinations of their values. As usual, the solution was found in their unification in the form of indices. Analyzing the results of studies on the relationship between the weight of the structures of the quail egg contents, in particular the weight of the yolk, *W_y_*, relative to the complex of parameters *W*, *V* and *S*, Narushin et al. [188] drew attention to the following empirical dependence:

(33) $$W_{y}=594.675W^{-3.325}V^{7.452}S^{-4.623},$$

where *W_y_* and *W* are measured in g, *V* in cm^3^ and S in cm^2^.

The above formula, taking into account some rounding of degrees, can be transformed into the following one:

(34) $$W_{y}=594.675\frac{V^{3}}{W^{3}}\cdot \frac{V^{4.5}}{S^{4.5}}.$$

### 8.2. Three Indexed Derivates and Prospective DL Applications

The resulting form of Equation (34) allows the assumption that instead of the parameters *W*, *V* and *S*, their mathematical derivatives, i.e., egg *density* (*D* = *W*/*V*) and metabolism index (*S*/*V*), can be used. As a result, the new empirical dependence derived was practically no different in accuracy from Equation (34):

(35) $$W_{y}=\frac{35.166}{D^{0.014}{\left(\frac{S}{V}\right)}^{2.984}},$$

with *R*^2^ = 0.454 (*p* < 0.01).

As it turned out, a similar approach worked when studying the quality indicators of quail eggshells [189], by which the authors used shell strength, *F*, thickness, *T*, and weight, *W_s_*. In addition to the parameters *W*, *V* and *S*, Narushin et al. [189] used the value of shell deformation under the impact of a non-destructive load of 0.5 kg (*δ*_0.5_). As a result, the following equations were obtained:

(36) $$F=0.000076\frac{{\left(\frac{S}{V}\right)}^{2.746}}{{\left(\frac{\delta_{0.5}}{W}\right)}^{1.62}},$$

(37) $$T=\frac{0.244}{{\left(\frac{\delta_{0.5}}{W}\right)}^{0.051}{\left(\frac{S}{V}\right)}^{0.279}},$$

(38) $$W_{s}=\frac{1.499}{{\left(\frac{\delta_{0.5}}{W}\right)}^{0.092}{\left(\frac{S}{V}\right)}^{1.128}}$$

in which *F* is measured in kg, *T* and *δ*_0.5_ in mm, *W_s_* and *W* in g, *V* in cm^3^ and *S* in cm^2^.

In addition to the already known metabolism index (*S*/*V*), Narushin et al. [189] introduced another one, *δ*_0.5_/*W*, that was coined the *specific elastic shell deformation*.

Thus, in addition to the three key parameters of an egg (*W*, *V* and *S*), we can also talk about three key indices, i.e., *D*, *S*/*V* and *δ*_0.5_/*W*, which can be effectively used for the non-destructive prediction of egg contents or shell quality indicators.

How can one use the above indices or derived dependencies for implementation in the DL process? The prospects are quite encouraging. Suppose the task is to train AI to recognize eggs with a stronger shell. How feasible is this? Based on the produced mathematical dependence (36), this quality category includes eggs with a numerator value higher than average and, accordingly, a lower denominator value. The numerator is the metabolism index, *S*/*V*, the increased values of which, according to previously cited studies [130], are inherent in eggs of a more elongated, conical shape. However, the index of specific elastic shell deformation, *δ*_0.5_/*W*, should be lower, which may be influenced by a higher egg weight. Thus, by choosing the appropriate images of eggs from this quality category, it is possible to train AI in their efficient recognition. A similar situation is possible if there is a practical interest in eggs with a higher yolk content. Based on Equation (35), the *S*/*V* value should tend to decrease; this effect is present in more rounded eggs [130]. That is, in this case, the choice of appropriate egg images should be conducted taking this premise into account.

## 9. Conclusions and Remarks

The field of egg production in the poultry industry, as well as the entire field of oological science (e.g., for conservation purposes), stands to benefit, like many academic fields (and many walks of life, in fact), from the use of AI, ML, NNs and DL. A plethora of methodological approaches, analytical capabilities and theoretical bases have been accumulated, enabling us to perform experiments of any degree of complexity and production focus, as well as enabling the practical implementation of the results obtained. The first stage that contributes to this implementation is to teach AI to act in accordance with distinct and sophisticated algorithms aimed at fulfilling the tasks of further research. In addition, one of the exceptional capabilities of AI is in dealing with large volumes of data. The effective analysis of such volumes improves the accuracy and reliability of models. The ability of AI to take into account many factors simultaneously makes it an indispensable tool for multidimensional tasks.

The purpose of this review is to be an invaluable resource in elucidating the most important prospective areas for effective DL implementation, which can be summarized as follows:

1. *Pre-incubation egg sorting*. Why incubate eggs that will not hatch? Or eggs that will hatch, but are of the unwanted sex? It is much more effective to cull such eggs before putting them into the incubator, even if this is not at 100% efficiency. After all, even a partial reduction in the load on incubator stations promises considerable benefits.
2. *Optimization of egg storage periods*. This is one more application area where some eggs can be stored longer than others. At the same time, there are many eggs whose use in the further technological chain is advisable to be earlier than planned in accordance with the existing regulations. Thus, separating eggs (before placing them in storage) into groups in accordance with the predicted permissible shelf life can be an effective technological method that allows for an increase in shelf life.
3. *Optimization of incubation regimes*. Differences in the morphological parameters of eggs and unified incubation technology often lead to a decrease in hatchability. Owing to the separation of eggs into groups depending on shell thickness, yolk weight or the *S*/*V* ratio, and the corresponding choice of specific features of the incubation regime, this will increase the return on such technology.
4. *Index representation of the dimensional egg characteristics*. This will contribute to DL efficiency, as it enables this process to become more narrowly focused, i.e., concentrated on sorting images depending on combinations of several parameters. In order to increase the index information content for the possible practical use of the experimental results, it is necessary to unify these indices. In the category of geometric ones, it is proposed to pay close attention to the indices shape (*B*/*L*), asymmetry or displacement (*w*/*L*) and conicity (*D_p_*/*B*), as they can be prospective egg quality indicators for use in further research and practice. At the same time, it is advisable to not only study the relationship between the effectiveness of some technological operations with individual indices but also with their combinations, for example, the correlation of the EGI (“symbiosis” of the shape and conicity indices) with egg hatchability. As a complex index representation, one can use mathematical models describing the egg profile geometry, including all of the above indices. Herewith, in the process of analyzing the experimental results, it makes sense to compare the actual egg profile with a standard one. As such a standard, one can rely on a certain geometric object, e.g., a classical ovoid that is maximally close to the profile of an actual egg being evaluated, or on a certain known solid of revolution, e.g., an ellipsoid or a sphere. If a connection between the geometric features of an egg and the sex of the embryo really does exist, such an approach to assessing deviations in the egg profile can undoubtedly prove effective.

In addition to the so-called geometric indices, it is apparent that there is a whole set of others that can be conventionally called morphometric. The research results reviewed here demonstrate the extraordinary potential of the metabolism index, *S*/*V*. With its help, it is possible to sort eggs according to their potential for storage duration, especially if we also take into account the size of the air cell. This index is also part of a complex model of indices for a possible prediction of the weight of internal egg structures, which is also important for developing individual, or rather grouped, technological methods in incubation. Moreover, using the *S*/*V* index, it is possible to attempt the synchronization of fertile eggs in terms of the time of hatching chicks.

The “symbiosis” of various egg quality indices has proven to be invaluable information for timely decision making. The combination of egg indices has a synergistic information effect and, therefore, studies aimed at sorting eggs according to certain indices, further subdivided into subgroups according to others, may be much more effective. As this review has demonstrated, geometric indices have proven to be very effective along with the index, for instance, characterizing the specific size of the air cell, *V_ac_*/*W*, or the specific elastic shell deformation, *δ*_0.5_/*W*.

In summary, the discipline of bird egg research, colloquially named “Eggology” [6], is on the threshold and starting point of a stage of great scientific discoveries, technological achievements and industrial successes that will be feasible due to AI and, specifically, DL development. Decades of painstaking work lie ahead, the results of which will ensure its systematic, deep and targeted training and implementation for exploring and improving egg quality characteristics using non-invasive methodologies.

## Figures and Tables

**Table 1:** Basic terminology used.

Abbreviation	Terms and Definitions	References
**AI**	**Artificial intelligence**, referring to the intelligence displayed by machines, especially computer systems, is a branch of computer science that creates and examines tools and software that allow machines to sense their environment and use intelligence and learning to take actions that increase the likelihood that they will accomplish predetermined objectives.	[26,27]
**ML**	**Machine learning** is a branch of AI that focuses on creating and analyzing statistical algorithms that can learn from data and extrapolate them to new data, completing tasks without direct guidance.	[28,29]
**NN**	**Neural network**, also known as artificial neural network or neural net, is a model used in ML that is based on the architecture and operation of biological neural networks found in animal brains.	[30,31]
**DL**	**Deep learning** is a sub-branch of ML that focuses on using NNs to carry out classification, regression, representation learning and tasks by learning from data.	[32,33]

## Data Availability

Not applicable.
